# Ferulic Acid Protects Hyperglycemia-Induced Kidney Damage by Regulating Oxidative Insult, Inflammation and Autophagy

**DOI:** 10.3389/fphar.2019.00027

**Published:** 2019-02-05

**Authors:** Sayantani Chowdhury, Sumit Ghosh, Abhishek Kumar Das, Parames C. Sil

**Affiliations:** Division of Molecular Medicine, Bose Institute, Kolkata, India

**Keywords:** diabetes, kidney, oxidative stress, inflammation, autophagy, apoptosis, ferulic acid

## Abstract

Oxidative insult, inflammation, apoptosis and autophagy play a pivotal role in the etiology of diabetic nephropathy, a global health concern. Ferulic acid, a phytochemical, is reported to protect against varied diseased conditions. However, the ameliorative role and mechanisms of ferulic acid in averting STZ-mediated nephrotoxicity largely remains unknown. For *in vivo* study, a single intraperitoneal injection of streptozotocin (50 mg kg^-1^ body wt.) was administered in experimental rats to induce diabetes. The diabetic rats exhibited a rise in blood glucose level as well as kidney to body weight ratio, a decrease in serum insulin level, severe kidney tissue damage and dysfunction. Elevation of intracellular ROS level, altered mitochondrial membrane potential and cellular redox balance impairment shown the participation of oxidative stress in hyperglycemia-triggered renal injury. Treatment with ferulic acid (50 mg kg^-1^ body wt., orally for 8 weeks), post-diabetic induction, could markedly ameliorate kidney injury, renal cell apoptosis, inflammation and defective autophagy in the kidneys. The underlying mechanism for such protection involved the modulation of AGEs, MAPKs (p38, JNK, and ERK 1/2), NF-κB mediated inflammatory pathways, mitochondria-dependent and -independent apoptosis as well as autophagy induction. In cultured NRK-52E cells, ferulic acid (at an optimum dose of 75 μM) could counter excessive ROS generation, induce autophagy and inhibit apoptotic death of cells under high glucose environment. Blockade of autophagy could significantly eradicate the protective effect of ferulic acid in high glucose-mediated cell death. Together, the study confirmed that ferulic acid, exhibiting hypoglycemic, antioxidant, anti-inflammatory, anti-apoptotic activities and role in autophagy, could circumvent oxidative stress-mediated renal cell damage.

## Introduction

Diabetes mellitus has become a global health concern. The disease occurs due to depressed insulin secretion or reduced tissue sensitivity to insulin. Uncontrolled hyperglycemia in diabetic patients is associated with diabetic nephropathy, cardiomyopathy, retinopathy, etc. Diabetic nephropathy is a prevalent complication connected with type-1 as well as type-2 diabetes which often leads to end-stage renal failure in almost about 30% of diabetic individuals (Barr, 2001; Yan et al., 2007; Fioretto and Mauer, 2010; Tervaert et al., 2010). The complication is characterized by glomerular hypertrophy, podocytopenia, thickened basement membrane, fibrosis and increased extracellular matrix protein synthesis (Fioretto and Mauer, 2010; Tervaert et al., 2010; Kanwar et al., 2011). Sustained hyperglycemia is not only associated with excessive generation of free radicals but also impairs the intracellular anti-oxidative machinery which leads to oxidative stress. Of the multifactorial etiology of diabetic nephropathy, oxidative stress is cited as one of the leading factors which can activate NF-κB. This molecule in turn, controls the expression of a cascade of proinflammatory molecules. The upregulation of these signaling pathways contributes to the progression of diabetic nephropathy (Ghosh et al., 2009; Navarro-González et al., 2011). Furthermore, contribution of increased oxidative stress in modulating autophagic activity have been reported to be associated with the development of diabetic nephropathy (Yamahara et al., 2013; Ding and Choi, 2015). Autophagy, a highly conserved “self-eating” biological process (which involves the degradation and recycling of intracellular macromolecules and organelles) serves as a pro-survival mechanism from yeast to mammals. At a basal level, the process occurs constitutively and serves as an essential housekeeping mechanism to maintain cellular homeostasis of glomeruli and tubules under oxidative stress induced hyperglycemia (Yamahara et al., 2013; Ding and Choi, 2015). Under high-glucose environment, basal autophagy is significantly inhibited in cultured podocytes. Increased glucose concentration interferes with autophagy by down-regulating the expressions of Beclin-1, LC3 and Atg12-5, thereby disrupting the function of filtration barrier in podocytes (Fang et al., 2013). Studies suggest that the progression of diabetic nephropathy can be diminished significantly by suppressing oxidative insult.

In search of new therapeutic antioxidants for combating diabetic nephropathy, a ubiquitous phenolic compound namely ferulic acid can be a possible example. It is obtained from rice, wheat, oat, pineapple, coffee seed, artichoke, peanut etc (Kumar and Pruthi, 2014). Consumption of these food results in daily intake of 150–200 mg of ferulic acid (Gohil et al., 2012). The study of the beneficial effects of ferulic acid has only been accelerated after its biosynthetic pathway in plants and process of extraction from wheat and maize bran were investigated in detail (Shahidi and Naczk, 2003; Buranov and Mazza, 2009; Ghosh et al., 2017). The molecule possesses several protective activities such as antidiabetic, antioxidative, anti-inflammatory, anti-carcinogenic, hypotensive, hypolipidemic effects, etc. (Yagi and Ohishi, 1979; Balasubashini et al., 2003; Ohnishi et al., 2004; Srinivasan et al., 2007; Chowdhury et al., 2016a; Ghosh et al., 2017). Ferulic acid supplementation increases cellular antioxidant activities, thereby neutralizing ROS under pathophysiological condition (Srinivasan et al., 2007) as well as reduces the risk of coronary artery diseases by scavenging free radicals (Bourne et al., 2000). In Japanese oriental medicine, ferulic acid is used to combat inflammation. The molecule provided protection against drug-induced liver damage (Wu et al., 1995). Furthermore, ferulic acid has been reported to play an ameliorative role in autophagy by upregulating the downregulated expression of autophagic markers viz. LC3-II in both HeLa cells and primary hepatocytes under nutrient-rich medium (Bian et al., 2013).

Based on the limited knowledge of the protective effects of ferulic acid against diabetic nephropathy in the available literature, the field attracts special attention (Choi et al., 2011; Ghosh et al., 2017). Thus, the present study emphasizes on the molecular mechanism through which ferulic acid protects against hyperglycemia-induced oxidative stress-mediated renal dysfunction. For such a detailed mechanistic study, rats and NRK-52E cells were used as models. Our findings suggest that ferulic acid could significantly ameliorate oxidative stress-induced renal tissue impairment and apoptosis by inhibiting ROS generation, NF-κB activation, stress signaling pathway (p38, JNK, ERK 1/2) activation and by promoting autophagy. Hence, the study reveals the molecular mechanisms by which ferulic acid exerts its protection against hyperglycemia-induced renal dysfunction under diabetic pathophysiology.

## Materials and Methods

### Chemicals

Streptozotocin (STZ), 3-(4,5-dimethylimidazole-2-yl)-2,5-diphenyltetrazolium bromide (MTT), bovine serum albumin (BSA) and other chemicals used for experimental purposes were obtained from were obtained from Sisco Research Laboratory (Mumbai, India). Ferulic acid, fluorescein isothiocyanate/ FITC-conjugated Annexin V detection kit, 2′,7′-dichlorodihydrofluorescein diacetate (H_2_DCFDA), DHE, RNaseA, 3-Methyladenine (autophagy inhibitor), In Situ Cell Death Detection Kit, Fluorescein (Roche) were obtained from Sigma-Aldrich Chemical Company (St. Louis, MO, United States). Bicinchoninic acid assay kit (BCA), Halt Protease and Phosphate Inhibitor Cocktail, DAPI, TRIzol reagent and Thermo Scientific Verso cDNA synthesis kit were purchased from Thermo Fisher Scientific Inc. (United States). HRP/DAB detection IHC kit was obtained from Abcam (Cambridge, United Kingdom). Kits for measuring parameters such as blood glucose (Advanced Accu-check glucometer), insulin (ELISA), blood glucose nitrogen, etc. were purchased from RayBiotech, Roche, Abcam and Span Diagnostic Ltd. (Gujarat, India). Dulbecco’s modified Eagle’s medium (DMEM), trypsin, non-essential amino acids, sodium bicarbonate, L-glutamate, glucose and sodium pyruvate were purchased from HIMEDIA (Mumbai, India) whereas; fetal bovine serum (FBS) was purchased from Gibco, Invitrogen (Carlsbad, CA, United States). Antibodies were obtained from Cell Signaling (Cell Signaling Technology Inc., Danvers, MA, United States), Abcam (Cambridge, United Kingdom), Novus Biologicals (Centennial, CO, United States). Precision Plus_TM_ Protein Dual Color Standards and were purchased from Bio-Rad Laboratories (Hercules, CA, United States).

### Animals

Male Wistar rats (adult, healthy of approximately 180–200 g weight) were acclimatized before the experiment under optimum conditions of the laboratory for 2 weeks and were sustained with standard water and standard pellet diet (Agro Corporation Private Ltd., Bangalore, India). The animals were exposed to a temperature of 25 ± 2°C and humidity of 30 ± 10% and alternating 12 h light and dark cycles. All the experiments were conducted following the guidelines and regulations of the IAEC (Institutional Animal Ethics Committee), Bose Institute, Kolkata (the permit number-IAEC/BI/3(I) cert./2010) and the study was approved by both CPCSEA (Committee for the Purpose of Control and Supervision on Experiments on Animals), Ministry of Environment and Forests, New Delhi, India (the permit number-1796/PO/Ere/S/14/CPCSEA) and IAEC.

### Determination of Free Radical Scavenging Activity of Ferulic Acid

Following the method of Blois (Blois, 1958), DPPH radical scavenging capacity of ferulic acid was assessed in a cell-free environment. Precisely, 1 ml of DPPH solution (125 μM) was mixed with 1 ml of test samples having different concentrations of ferulic acid (0–200 μM) in separate tubes followed by incubation of the solutions in the dark at 37°C for 30 min. The absorbance was measured spectrophotometrically at 517 nm against methanol blank. DPPH radical scavenging ability was determined in terms of percentage of DPPH radical scavenged in respect to the control (Ascorbic acid: positive control).

### Assessment of Antioxidant Activity (Total) of Ferulic Acid

The method of Benzie and Strain with some minor modifications (Benzie and Strain, 1996; Sinha et al., 2015) was adapted to determine the ferric reducing antioxidant effect (FRAP assay) of ferulic acid. Briefly, in a cell-free system, different concentrations of ferulic acid (0–100 μM) were permitted to react with freshly prepared FRAP solution (300 mM acetate buffer, pH 3.6; 10 mM TPTZ; 20 mM FeCl_3_.6H_2_O in the ratio 10:1:1 respectively) and incubated for 30 min at 37°C in the dark. The increase in the absorbance was measured spectrophotometrically at 595 nm. Ascorbic acid was used as the positive control.

### Diabetes Induction in Experimental Rats

**FIGURE 1:**
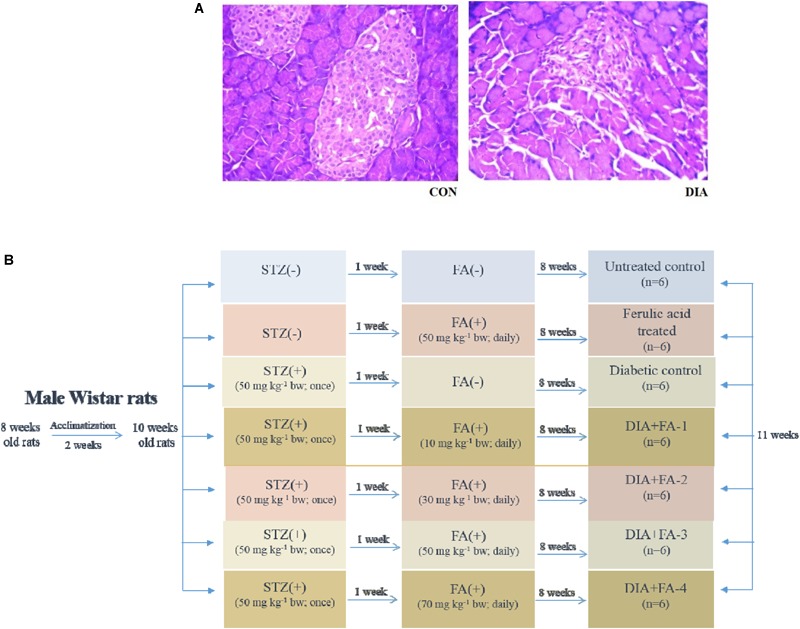
Validation of diabetes induction and schematic representation of *in vivo* experimental design. **(A)** Hematoxylin-eosin staining of sections of rat pancreas (×200); CON: received only vehicle, i.e., water; DIA: a single dose of STZ was given (50 mg kg^-1^ body wt., intraperitoneally). The non-diabetic animals showed a regular healthy pancreas structure whereas; the pancreas of diabetic rats showed degeneration as well as shrinkage of islets, thereby confirming diabetes induction; **(B)** *in vivo* study design.

Induction of diabetes was carried out in the experimental animals (after overnight fasting) with a single intraperitoneal injection of STZ (50 mg kg^-1^ body wt., prepared in 0.1 M citrate buffer, pH 4.5) (Aziz et al., 2012). After 7 days of the administration of STZ, Advanced Accu-check glucometer was used to measure the glucose levels in the blood of overnight fasting rats. The experimental animals with blood glucose level ≥ 280 mg/dL were classified as diabetic and used for further experiments. Histological assessment of the pancreas was also carried out to validate diabetic induction in the experimental rats (Figure 1A).

### Determination of Dose-Dependent and Time-Dependent Role of Ferulic Acid by Measuring the Glucose Level in Blood and BUN Assay

A dose-dependent and time-dependent study was conducted to obtain the optimum dose of ferulic acid and the same was selected by measuring the fasting blood glucose and BUN levels. The experimental rats were randomly divided into six groups and each group comprised of six animals. Of these groups, two groups functioned as (i) controls, receiving only the vehicle and (ii) the diabetic group, receiving STZ (a single dose of 50 mg kg^-1^ body wt, i.p.). The remaining four sets were administered with ferulic acid of varied doses (10, 30, 50, and 70) mg kg^-1^ body wt. (in distilled water, post-diabetes, orally, daily) for 8 weeks which is by the previous study conducted by Chowdhury et al. (2016a).

The experimental set up for the *in vivo* study has been shown in Figure 1B.

The effective dose of ferulic acid was determined by observing the effect of the same on both fasting blood glucose and BUN levels. Oral administration of ferulic acid (50 mg kg^-1^ body wt.), post-diabetes, for 8 weeks has been considered to be the optimum dose which effectively ameliorated the above mentioned altered parameters. Beyond the above mentioned effective dose and treatment period, ferulic acid, however, did not impart any additional benefit as compared to the used procedure.

### Experimental Design (*in vivo*)

Experimental animals were randomly divided into four groups after the optimum effective dose of ferulic acid was determined.

Group 1: (CON): comprised of 6 experimental rats receiving only water as vehicle.

Group 2: (FA-3): comprised of 6 experimental rats receiving an oral administration ferulic acid, daily for 8 weeks (dose: 50 mg kg^-1^ body wt.).

Group 3: (DIA): comprised of 6 experimental rats receiving a single intraperitoneal dose of STZ (50 mg kg^-1^ body wt.).

Group 4: (DIA + FA-3): comprised of 6 experimental rats receiving ferulic acid (dose: 50 mg kg^-1^ body wt.), after diabetic induction, orally for 8 weeks.

### Blood, Urine and Kidney Collection

Following 8 weeks of treatment with ferulic acid, all the experimental rats were sacrificed followed by the collection of blood and urine. Both the plasma and the serum were collected from blood samples of the experimental rats. Kidneys were fixed in 10% buffered formalin for histological assessments and stored at -80°C until further use.

### Determination of Biochemical Parameters

#### Renal and Hepatic Biomarkers

Activities of hepatic enzymes viz. SGPT and SGOT together with creatinine, BUN, uric acid and urinary albumin/urinary creatinine ratio were determined using commercially available standard kits.

#### Assay of Enzymatic and Non-enzymatic Antioxidants

##### Determination of glutathione S-transferases activity

Twenty five μg of protein obtained from each kidney tissue homogenate was mixed with potassium phosphate buffer, EDTA, CDNB, and GSH at 37°C following the protocol of Habig et al. (1974) and the increase in absorbance of the conjugate of CDNB and GSH was measured at a wavelength of 340 nm. One unit of GST activity is equivalent to 1 μmol of the product formed min^-1^.

##### Measurement of glutathione peroxidase activity

Glutathione peroxidase activity was measured by adapting the method of Flohé and Günzler (1984). Briefly, NADPH and H_2_O_2_ were used as substrates and the changes recorded in the absorption intensity at 340 nm is proportional to the conversation of NAPDH to NADP^+^. One unit of GPx activity is termed as the amount of enzyme capable catalyzing the oxidation of 1 μmol of NADPH min^-1^.

##### Measurement of antioxidant enzymes’ activities

Activities of SOD, catalase, GR, GSH and GSSG in the renal tissue were measured based on the protocol followed by Chowdhury et al. (2016a).

#### Estimation of Glycosylated Hemoglobin, Lipid Peroxidation and Protein Carbonyl Content

The protocol of Nayak and Pattabiraman (1981) was followed to estimate the concentration of glycosylated hemoglobin (Hb).

Lipid peroxidation was determined by performing a colorimetric reaction assay with thiobarbituric acid (TBA) by adapting the protocol of Esterbauer and Cheeseman (1990). The absorbance of TBARS, i.e., TBARS was recorded at 532 nm and using the extinction coefficient of MDA (1.56 × 10^5^ M^-1^ cm^-1^), the concentration of TBARS, since 99% population of TBARS exists as MDA.

On the other hand, determination of protein carbonyl content was based on protein hydrazone formation following the reaction with 2,4-DNPH and the absorbance was measured spectrophotometrically (wavelength: 365 nm). The results were calculated as DNPH (expressed in terms of nmol) that were incorporated in mg^-1^ of protein (aliphatic hydrazones’ molar extinction coefficient: 22,000 M^-1^ cm^-1^) (Uchida and Stadtman, 1993).

#### Determination of Nitric Oxide (NO) Level

The nitrite levels in tissue samples were measured spectrophotometrically following the protocol based on Griess reaction (Beda and Nedospasov, 2005) which in turn assessed the NO concentration indirectly. Samples (20 μl) were added to 100 μl of Griess reagent [freshly prepared 2% sulfanilamide in 5% HCl and 0.1% *N*-(1-naphthyl) ethylenediamine dihydrochloride in H_2_O in the ratio 1:1], NADH and nitrate reductase. One milliliter of H_2_O was added, mixed and stirred for 5 min and the reading was taken at 550 nm using a spectrophotometer. NaNO_2_ was used as a standard for the experiment.

#### Determination of MPO Activity in the Kidney Tissue

The MPO activity was measured using 3,3′,5,5′-Tetramethylbenzidine (TMB) according to the protocol of Suzuki et al. (1983). Briefly, experimental samples (10 μl) were mixed with 0.75 mM H_2_O_2_ (80 μl) and TMB solution (110 μl) and incubated for 5 min at 37°C. The reaction was terminated using 2M H_2_SO_4_ (50 μl) and the absorbance was measured spectrophotometrically at 450 nm for the determination of the MPO activity (expressed regarding μM of H_2_O_2_ consumed min^-1^mg^-1^ of protein.

#### Measurement of Xanthine Oxidase Activity and Renal Hydroxyproline Level

Xanthine oxidase activity was evaluated by measuring the enzymatic oxidation of xanthine. Briefly, to 100 μl of kidney tissue homogenate, 1.9 mL of potassium phosphate buffer (50 mM, pH 7.5) and 1 mL of xanthine (0.15 mM) were added. The absorbance was measured spectrophotometrically for 4 min (wavelength: 290 nm) (Pal et al., 2014).

The level of hydroxyproline was calculated following the protocol of Woessner (1961).

### Histological Studies

Kidneys of rats from different experimental groups were collected, fixed, processed and paraffin sectioned. H&E staining protocol was used to stain the tissue sections of 5 μm thickness and observed under light microscope. The average glomerular volume was taken into consideration to assess parameters such as glomerulosclerosis and tubulointerstitial damage and the mean values were calculated (each of six glomeruli per section). For quantification of renal tissue damage, the histologic scoring system was used where it indicated the following: “0”- absent; “1”- present; and “2”- marked.

### Detection of Intracellular ROS Production

Intracellular ROS production was measured by using DCFDA as a probe following the protocol of LeBel and Bondy (Rashid et al., 2017) with few modifications (Chowdhury et al., 2016b). Briefly, in 100 μl of kidney tissue homogenates, were incubated with 1 ml of assay media (20 mMTris-HCl, 130 mMKCl, 5 mM MgCl_2_, 20 mM NaH_2_PO_4_, 30 mM glucose, and 5 μM DCFDA) for 15 min at 37°C. On the other hand, *in vitro*, adopting the protocol of Cossarizza et al. (2009), cells were exposed to glucose and ferulic acid at the above mentioned dose and specified time. Following incubation, cells were scrapped followed by centrifugation (at 300 *g* for 5 min at room temperature) and the pellets thus obtained were suspended in 1ml of PBS and H_2_DCFDA (having a final concentration of 2 μM) was added. The cells were incubated for 20 min at 37°C in the dark followed by FACS analyses. For both *in vivo* and *in vitro* samples, DCF formation was measured using FITC filters equipped fluorescence spectrometer (FACSVerse, Hitachi) (excitation/emission: 488/520 nm) for 10 min (Rashid et al., 2017) and analyzed by FACSuite software.

On the other hand, ROS generation (intracellular) was quantified by using the oxidative fluorescent dye namely, DHE (extensively used to monitor superoxide radical production). Cryosections of renal tissues of rats from different experimental sets (10 μm) were stained with 10 μmol/L of DHE and incubated in a humidified chamber for 15 min in the dark at 37°C and observed under a confocal microscope (Chowdhury et al., 2016b).

### Renal Tissue Homogenate Preparation

The kidneys, collected from experimental sets were minced and washed in phosphate-buffered saline PBS (1X) followed by homogenization in protease and phosphatase inhibitors supplemented cold radioimmunoprecipitation assay (RIPA) lysis buffer, 1:3 (w:v), [composition: 150 mM sodium chloride, 0.1% sodium dodecyl sulfate (SDS), Triton X-100, 50 mM Tris, 0.5% sodium deoxycholate, pH 8.0] in a Dounce glass homogenizer. The homogenates, thus obtained, were centrifuged at 12,000 rpm for 10 min at 4°C and subsequently aliquoted followed by storage of the same for further experiments at -80°C.

### Preparation of Subcellular Fractions of Kidney Tissue to Obtain Cytoplasmic, Mitochondrial, and Nuclear Fractions

The protocol of Cox and Emili (2006; Rashid et al., 2017) (slightly modified) was implemented to obtain the subcellular fractions. The kidney samples were washed in PBS, homogenized in protease and phosphatase inhibitors supplemented cold buffer namely 250-STMDPS (50 mM Tris-HCl having a pH of 7.4, 5 mM MgCl_2_, 25 μg ml^-1^ spermidine, 250 mM sucrose, 1 mM DTT and 1 mM PMSF) to which protease and phosphatase inhibitors were added and centrifuged (800 *g*, 4°C, 15 min). To isolate the nuclear fraction, the supernatant, thus obtained, i.e., Sup I was kept aside and 250-STMDPS buffer were added to the pellet, homogenized and centrifuged (800 *g*, 4°C, 15 min). The pellet was suspended in 5 volumes of NET buffer (20 mM HEPES having a pH of 7.9, 0.5 M NaCl, 1.5 mM MgCl_2_, 0.2 mM EDTA, 1% Triton-X-100, 20% glycerol, 1 mM DTT and 1 mM PMSF) containing protease and phosphatase inhibitors followed by an incubation for 30 min, occasional vortexing at 4°C, lysis by sonication and centrifugation (14000 *g*, 4°C, 25 min). The obtained supernatant was for immunoblot concerning Lamin B1 and NF-κB p65. To separate the mitochondrial fraction, Sup I was centrifuged (4°C, 6000 *g*, 15 min). The obtained supernatant, i.e., Sup II was kept aside and the pellet was suspended in five volumes of buffer ME (20 mM Tris-HCl having a pH 7.8, 15% glycerol, 0.4 M NaCl, 1.5% Triton-X-100, 1 mM DTT and 1 mM PMSF) supplemented with protease and phosphatase inhibitors to obtain the mitochondrial fraction, lysed (by sonication) and centrifuged (4°C, 14000 *g*, 15 min). The obtained supernatant was for immunoblot concerning VDAC and cytochrome c. To obtain the cytoplasmic fractions, Sup II was centrifuged (100,000 *g*, 4°C, 60 min), collected and used for Western blotting concerning β-actin and cytochrome c.

The purity of subcellular fractions was checked through immunoblot where VDAC and Histone H3 were used as marker proteins for the mitochondrial and nuclear fractions of the kidney tissue respectively from the control group (data not shown).

#### Estimation of Protein Content and Immunoblotting

The experimental samples were measured for the protein content using the BCA assay kit.

An equal amount of protein from the lysate of each experimental sample was resolved by SDS-PAGE (10–12%; as required) for western blot analysis (Chowdhury et al., 2016b). The following primary antibodies (each having a dilution of 1:1000) were used: anti-AGE, anti-p-NF-κB p65, anti-phospho-IκBα, anti-IκBα, anti-phospho ERK1/2, anti-ERK1/2, anti-caspase-3, anti-PARP, anti-VDAC, anti-β-actin, anti-Lamin B1, anti-Cox-2, anti-iNOS, anti-phospho-p38, anti-p38, anti-phospho-JNK (1:1000), anti-JNK, anti-cytochrome c, anti-Bcl-2, anti-Bax, anti-caspase-8, anti-caspase-9, anti-beclin 1, anti-LC3B and anti-p62. HRP-conjugated secondary antibody (dilution of 1:20,000) was used to detect the primary antibodies using ECL solution (HRP substrate).

### RNA Extraction and Real-Time/RT-PCR

Total RNA was extracted from the kidney tissues of all the experimental groups using TRIzol reagent, RNA concentration was measured (Nanodrop, HellmaTrayCell Type 105.810) and verso cDNA synthesis kit was used to prepare cDNA from 1 μg of total RNA. The following thermal cycling was performed: (95°C, 5 min) followed by a set of 35 cycles: (95°C, 30 s; T_m_ °C, 30 s and 72°C, 45 s) followed by DNA extension (72°C, 5 min). Real-time (RT) PCR was performed with 2 μl of diluted stock of cDNA (1:5) using SYBR green PCR system, in triplicate, on 7500 Fast (Applied Biosystems) (Mahata et al., 2015). mRNA quantification data were normalized to loading control, i.e., GAPDH and expressed regarding fold differences of target gene expression relative to the vehicle-treated group. RT primers have been listed below.

Primer Sequences (5′-3′)

TNF-α: (sense: CTGAAGTAGTGGCCTGGATTG, antisense: GCTGGTAGTTTAGCTCCGTTT)

IL-1β: (sense: CTTCCTAAAGATGGCTGCACTA, antisense: ATCCCATACACACGGACAAC)

IL-6: (sense: CAGAGCAATACTGAAACCCTAGT, antisense: TTCTGACCACAGTGAGGAATG)

MCP-1: (sense: GTGTCCCAAAGAAGCTGTAGTA, antisense: AAGGCATCACATTCCAAATCAC)

VCAM-1: (sense: GAGTGCAAGAAGCCAACTAGA, antisense: AGCTGCCTACTCAACATTAACA)

ICAM-1: (sense: CACCATGCTTCCTCTGACAT, antisense: CACTGCTCGTCCACATAGTATT)

FasR: (sense: TGTCAACCGTGTCAGCCTG, antisense: GTGCAAGGCTCAAGGATGT)

FasL: (sense: AATGCCTGCATCATGAGCCA, antisense: AGTCTCTAGCTTATCCATGA)

β-Actin: (sense: TCCCTGGAGAAGAGCTATGA, antisense: ATAGAGCCACCAATCCACAC)

### Mitochondrial Membrane Potential Determination

Mitochondria were isolated from kidney tissues of the experimental animals to determine the changes in MMP (ΔΨm). Cell-permeable fluorescent cationic dye, namely Rhodamine123 (Rh123) and FACScan flow cytometer were used to perform the analytic flow cytometric measurements of ΔΨm of isolated mitochondria and the measurement was done on the basis of cellular retention of the fluorescent cationic probe followed by analysis of the data using the Cell Quest software (Hodârnâu et al., 1973; Mingatto et al., 2002).

### Immunohistochemistry

Immunohistochemical staining for AGE was performed following the manufacturer’s protocol. Buffered formalin (10%) was used to fix the kidney tissues (isolated from the experimental groups) and subsequently processed for paraffin section. Sections of renal tissue (6 μm) were deparaffinized using xylene followed by rehydration with a down gradation of ethanol to water. The slides were subsequently subjected to the heat induced antigen retrieval protocol, i.e., by boiling samples in citrate buffer (0.1 M, pH 8.8) for 20 min in the microwave for an adequate signal. H_2_O_2_ (3%) was applied to block the endogenous peroxidase for 10 min and after that washed with 1 X TBS, incubated at 4°C, overnight with an anti-AGE antibody which was localized using a secondary antibody-HRP conjugate which reacted with the primary antibody and substrate-chromogen (DAB). Leica Microsystem DN1000 Microscope (camera: DFC450 C) was used to view the slides.

### Agarose Gel Electrophoresis for DNA Laddering and Detection of Apoptosis by TUNEL Assay

DNA fragmentation assay was performed following genomic DNA isolation on agarose/EtBr gel (Sellins and Cohen, 1987) and the DNA ladder was viewed on a UV transilluminator.

On the other hand, TUNEL assay was performed to quantify apoptosis on paraffin-embedded sections of tissue of experimental rats (Lespagnol et al., 2008) and analysis followed using In Situ Cell Death Detection Kit, Fluorescein (adapting manufacture’s protocol). Briefly, kidney tissues were dewaxed followed by rehydration and permeabilization and incubated with TUNEL reaction mixture (50 μl) in a humidified chamber in the dark for 1 h at 37°C. The sections were washed (1X PBS) and counterstained with DAPI (1 μg/ml) and incubated in the dark for 5 min, washed with PBS, mounted under a coverslip using antifade mounting media and examined under confocal microscopy (Leica).

### Evaluation of Ferulic Acid Absorption in Plasma

Blood samples from the experimental group treated with ferulic acid only were collected at different time points 0, 15, 30, 60, 180, 360, 720, and 1440 min after administration of ferulic acid for evaluation of its absorption. The samples were mixed with 4% trisodium citrate in the ratio 1:9, centrifuged at 5000 rpm for 15 min, filtered and injected directly into the column to detect the concentration of the molecule by the methods of HPLC. Ferulic acid concentration in the plasma was detected using a Shimadzu model LC20-AT pump system equipped with an analytical Phenomenex C18 reversed-phase column attached to a model SIL-20A auto-sampler and a diode array model SIL-M20A detector. A binary mixture of acetonitrile-water (16:84 v/v) containing glacial acetic acid (1%) was applied to mobile phase (flow rate of 1.0 ml min^-1^). Ferulic acid was eluted using a programmed gradient solvent system and the detection was made at 320 nm. The measurements were performed at room temperature. The data thus obtained was analyzed using the LCsolution Version 1.21 software (Ge et al., 2015; Sinha et al., 2015).

### Cell Culture and Treatment

Normal rat kidney epithelial-like cell line (NRK-52E cells) were cultured in DMEM supplemented with 10% FBS, 100 μg/ml streptomycin, 100 U/ml penicillin, 2.5 μg/ml amphotericin and 50 μg/ml gentamicin in a CO_2_ incubator at 37°C, maintaining CO_2_ at 5%. Cells were pre-treated with or without ferulic acid (0, 5, 25, 50, 75, 100, and 200 μM) for 2 h followed by 5.5 mM glucose (control glucose group) or 25 mM glucose (high glucose group) exposure for the next 48 h. Mannitol (19.5 mM) was used as osmolarity control (data not shown). The dose and the optimum time of incubation of glucose for NRK-52E cells were chosen according to a previous study (Hou et al., 2014; Wei et al., 2015), whereas, the dose of ferulic acid was selected from 0 to 200 μM. The experiments were conducted in serum-free medium under sterile conditions and performed in triplicate for better reproducibility.

Here, it is worth mentioning that, in this study, NRK-52E cells have been considered for autophagy inhibition study because it is a widely established supportive model (especially to support *in vivo* study) in prolonged high glucose-exposed renal cell damage (Hou et al., 2014).

#### Cell Viability Assessment

To assess the cell viability, NRK-52E cells were grown in 96 well plate and incubated with different concentration of ferulic acid and glucose 48 h. Following the incubation period, in each case, the media was discarded and the cells were washed gently with PBS twice. MTT assay was performed to measure the cell viability. The absorbance was measured at a wavelength of 570 nm (with background subtraction at a wavelength of 630 nm) (Sinha et al., 2015).

#### Assessment of Cellular Morphology of NRK-52E by Phase Contrast Microscopy

NRK-52E cells cultured in 6-well plates and were divided into control, high glucose (25 mM) exposed, 75 μM ferulic acid pre-treated + high glucose, only 75 μM ferulic acid treated and mannitol exposed groups. Following glucose exposure in presence or absence of ferulic acid for 48h, the medium was discarded and washed with PBS and successively viewed under phase contrast microscope (Leica DN1000; camera: DFC450 C).

#### Detection of NRK-52E Cell Death by Annexin V Affinity Assay

NRK-52E cells in 6-well plates and DMEM was replaced with the serum-free medium when the cells reached 80% confluency and were exposed to glucose and ferulic acid and the previously mentioned dose and time point. The cells were scrapped off gently the following incubation and centrifuged (300 g, 5 min, room temperature) followed by washing of the pellets with PBS and resuspension of the same in Annexin V affinity binding buffer (1 X). 1 ml of Annexin V/FITC was added to the samples and incubated for 5 min at room temperature in the dark followed by immediate analyses of the same using FACSVerse at excitation and emission wavelengths of 488 and 520 nm respectively (Sinha et al., 2014).

### Statistical Analyses

Results have been represented as mean ± SEM for three independent experimental sets. Statistical evaluation for every data was performed using ANOVA whereas; group means were compared by implying the Tukey method using Origin8 software (Originlab, Northampton, MA, United States). For real-time PCR data analysis, “paired *t*-test” was performed. A *p*-value < 0.05 was regarded to be significant statistically.

## Results

### Ferulic Acid Exhibits Free Radical Scavenging and Antioxidant Activities

**FIGURE 2:**
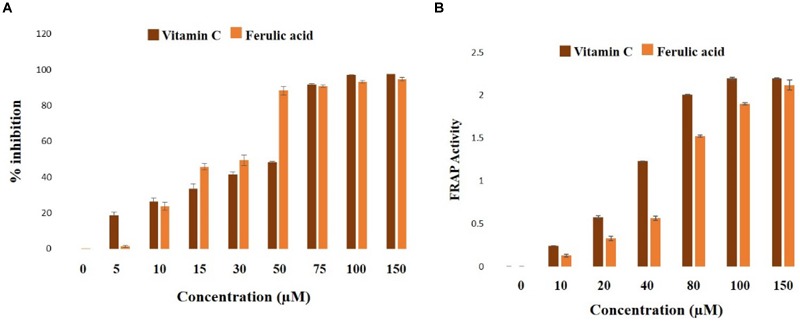
Biochemical properties of ferulic acid in the cell-free system. **(A)** Radical scavenging activities of DPPH (represented as the percentage of inhibition over control) of ferulic acid and Vitamin C; **(B)** FRAP assay depicting the ferric reducing capacity of ferulic acid and Vitamin C. Data are denoted as the mean ± SEM of three independent experiments.

2,2-diphenyl 1-picryl hydrazyl radical scavenging and FRAP activities of ferulic acid were assessed in a cell-free system. As observed from the data, ferulic acid could significantly (*p* < 0.05) scavenge DPPH free radical in a concentration-dependent manner showing IC_50_ at approximately 33 μM (Figure 2).

In FRAP assay, the molecule exhibited half of the maximum ferric reducing property at 38 μM (Figure 2B), thus exhibiting an excellent antioxidant property.

### Dose and Time-Dependent Role of Ferulic Acid on Blood Glucose and BUN Levels in STZ-Triggered Diabetic Animals

**FIGURE 3:**
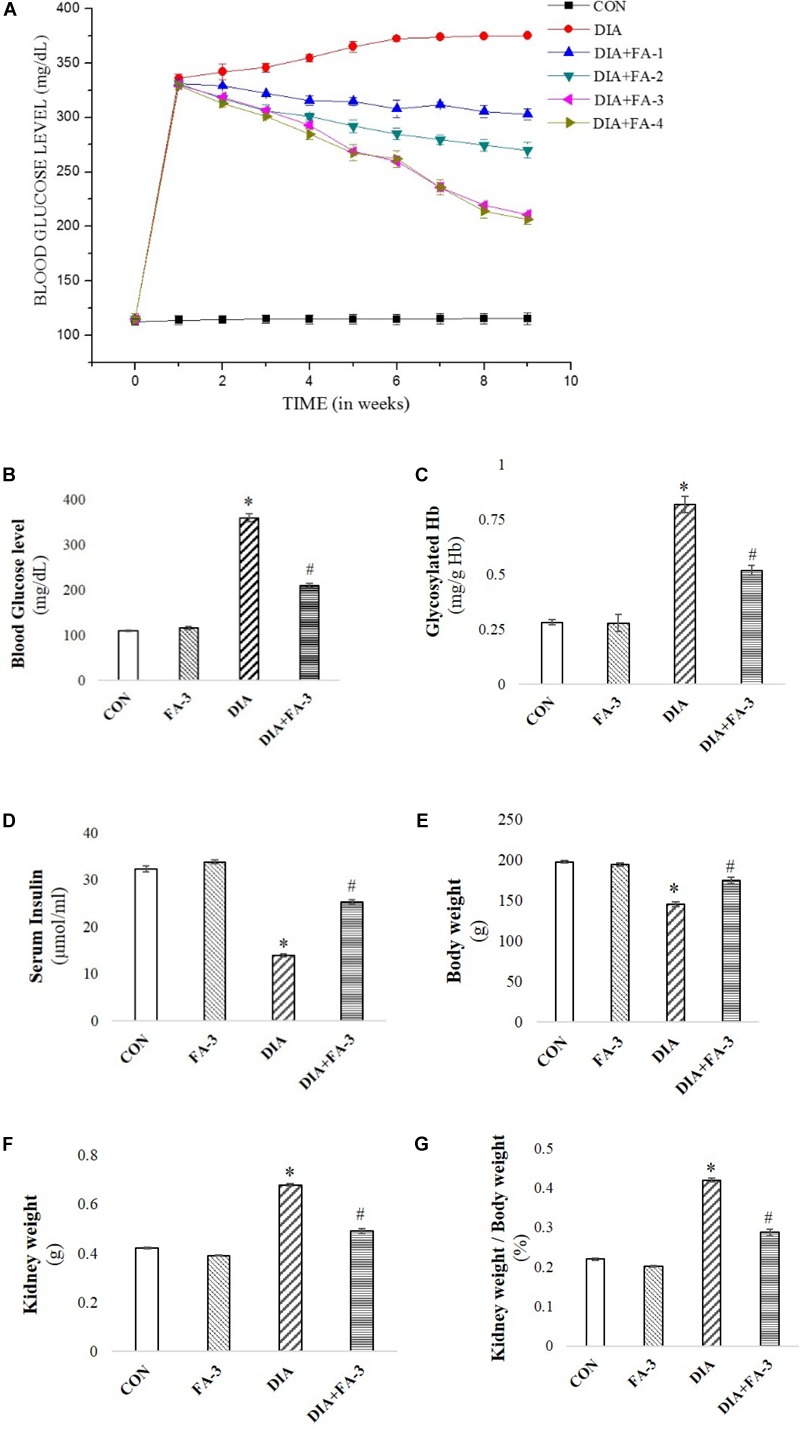
Effect of STZ and ferulic acid on the markers of the diabetic pathophysiology. **(A)** Effect of ferulic acid on blood glucose level of STZ-triggered diabetic rats in both dose-dependent and time-dependent manner. CON: level of blood glucose on untreated rats; STZ: blood glucose level of STZ-induced rats; DIA + FA-1, DIA + FA-2, DIA + FA-3, DIA + FA-4: the level of blood glucose of ferulic acid-treated diabetic rats at a dose of 10, 30, 50, and 70 mg kg^-1^ body wt. respectively for 8 weeks; **(B)** blood glucose level; **(C)** glycosylated Hb; **(D)** serum insulin level, **(E)** absolutebody weight of experimental rats, **(F)** absolute kidney weight, and **(G)** kidney-to-body weight ratio of experimental animals. CON: rats treated with vehicle only; FA-3: rats were subjected to only ferulic acid treatment at a dose of 50 mg kg^-1^; STZ: diabetic control; STZ + FA-3: diabetic rats were subjected to ferulic acid treatment at a dose of 50 mg kg^-1^. Values are represented as mean ± SEM (six animals in each experimental groups) for three independent experiments. “^∗^” symbolizes values differing from CON (^∗^*P* < 0.05) significantly; “#” denotes values differing from DIA (^#^*P* < 0.05) significantly; no significant variance existed between untreated (CON) and ferulic acid treated (FA-3) groups.

**FIGURE 4:**
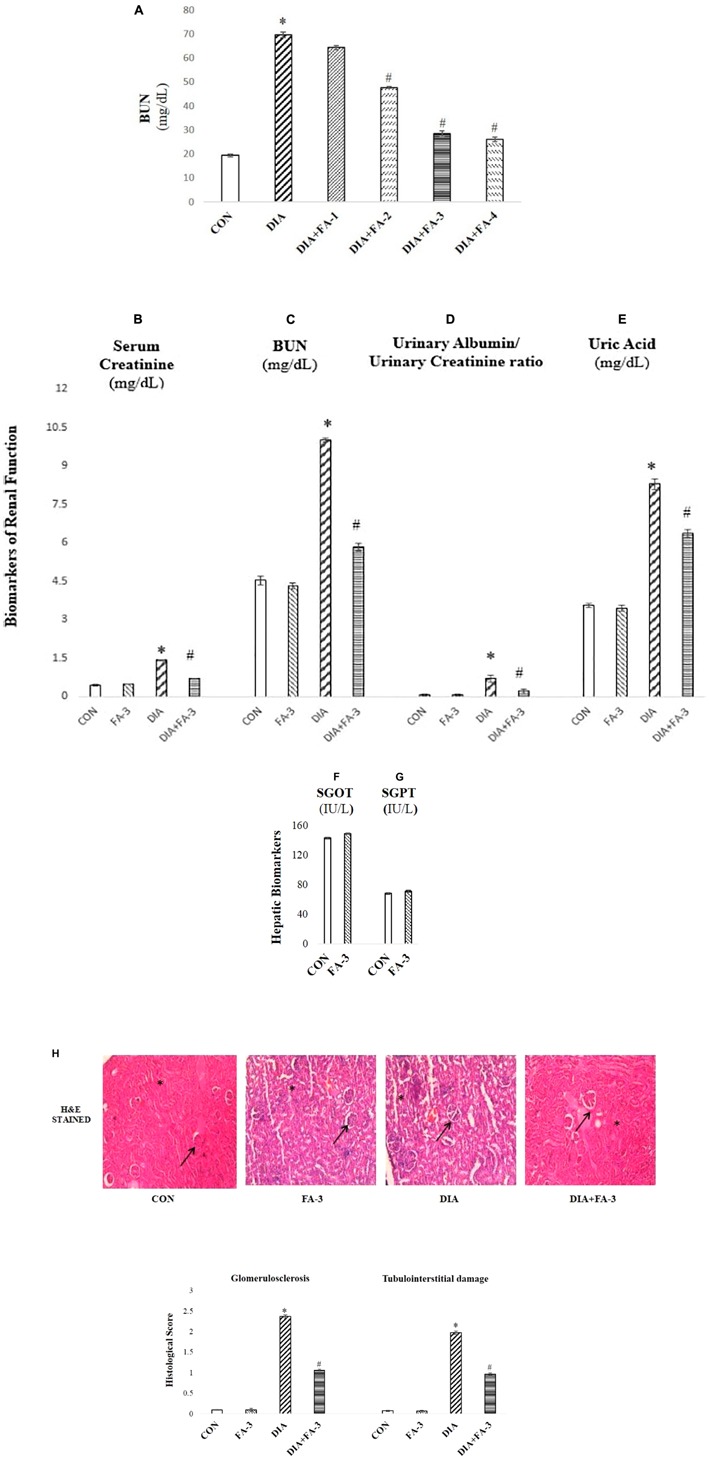
The role of ferulic acid on STZ-mediated nephrotoxicity in type 1 diabetic rats. **(A)** Effect of ferulic acid on the level of BUN in serum alongside STZ mediated toxicity in the renal tissue of the experimental rats in a dose-dependent manner. CON: BUN level in rats treated with vehicle only; STZ: BUN level in STZ induced diabetic rats; STZ+FA-10, STZ+FA-30, STZ+FA-50, STZ+FA-70: BUN level in ferulic acid treated diabetic rats for 8 weeks at varied doses viz. 10, 30, 50, and 70 mg kg^-1^ body wt. respectively; **(B)** serum creatinine level; **(C)** BUN level; **(D)** urinary albumin/urinary creatinine ratio; **(E)** level of uric acid; **(F)** SGOT level; **(G)** SGPT level; **(H)** Histological examination. H&E staining of sections of kidney tissues of rats; ×200 and histological score. CON: rats receiving vehicle only; FA-3: only ferulic acid treated rats (dose: 50 mg kg^-1^ body wt.); STZ: receiving STZ (50 mg kg^-1^ body wt.); STZ+ FA-3: post-diabetic induction, rats treated with ferulic acid (dose: 50 mg kg^-1^ body wt.). Values are represented as mean ± SEM (six animals in each experimental groups) for three different experiments. “^∗^” symbolizes values differing from CON (^∗^*P* < 0.05) significantly; “#” denotes values differing from DIA (^#^*P* < 0.05) significantly; no significant variance existed between untreated (CON) and ferulic acid treated (FA-3) groups.

To determine the role of the molecule on blood glucose level in diabetic animals, a dose-dependent and time-dependent experimental study were conducted (Figure 3A; *p* < 0.05). Following diabetes induction, post-treatment with 50 mg kg^-1^ body wt. of ferulic acid for consecutive 8 weeks could ameliorate the altered level of blood glucose in a dose and time-dependent way. Also, BUN assay was performed to assess the optimum effective dose at which the molecule could protect against STZ-exposed kidney tissue damage. Experimental data suggested that STZ-induced increased BUN level could be significantly circumvented following ferulic acid administration (50 mg kg^-1^ body wt. daily, orally for 8 weeks) (Figure 4A; *p* < 0.05). Ferulic acid, at a higher dose and treatment period, however, did not exhibit any additional benefit to the blood glucose and the serum BUN levels. Hence, 50 mg kg^-1^ body wt. of ferulic acid was considered as the optimum dose for the subsequent *in vivo* experiments.

### Evaluation of Induced Diabetes, Alteration of Kidney-to-Body Weight Ratio and the Hypoglycemic Activity of Ferulic Acid

Reduced body weight, increased intake of water, elevated blood glucose and Hb levels and reduced serum insulin are the key markers of diabetic pathophysiology (Rashid and Sil, 2015a,b). In the present study, diabetes in STZ-induced rats was assessed by a significant increase in the blood glucose level (Figure 3B), glycosylated Hb (Figure 3C) and absolute kidney weight (Figure 3F) whereas; decrease in serum insulin (Figure 3D) and body weight (Figure 3E). The kidney to the body wt. ratio increased significantly (diabetic nephropathy marker) (Figure 3G). However, treatment with ferulic acid significantly combated all the anomalies mentioned above without imparting any toxic effect in the untreated group (Figure 3A–F; *p* < 0.05), thereby suggesting that the molecule possesses hypoglycemic activity.

### Ferulic Acid Ameliorates Kidney Dysfunction in Diabetic Rats

To check the renal dysfunction in hyperglycemic animals, the classical biomarkers of diabetic nephropathy such as the serum creatinine, BUN, uric acid and urinary albumin/urinary creatinine ratio were measured. Post-treatment with ferulic acid significantly decreased (*p* < 0.05) STZ-induced elevated serum creatinine (Figure 4B), BUN (Figure 4C), urinary albumin/urinary creatinine ratio (Figure 4D) and uric acid levels (Figure 4E).

Furthermore, the profile of serum markers viz. SGOT (Figure 4F) and SGPT (Figure 4G) were measured to ensure that ferulic acid itself, at the dose mentioned above, did not impart any liver toxicity. No significant variation was observed between the untreated and only ferulic acid treated animals.

H&E staining of kidney tissue from STZ-mediated type1 diabetic rats showed pathologic changes in glomeruli which is by the existing literature (Figure 4H; Das and Sil, 2012). The untreated group exhibited normal renal histology pattern (histological score: 0). In hyperglycemic rodents, glomerular volume/hypertrophy increased (on an average of 40% from CON and FA-3 groups). Scarring of glomeruli (glomerulosclerosis; histological score 2.37) and tubulointerstitial damage was evident from degenerated and damaged tubules (histological score 1.97), whereas; treatment with ferulic acid significantly (*p* < 0.05) reduced glomerular volume (by about 35%, compared to the diabetic control group), glomerulosclerosis (histological score 1.06) and tubulointerstitial (histological score 0.96) damage in the diabetic animals. These findings suggested that ferulic acid could protect against renal injury and glomerular hypertrophy under diabetic condition.

### Plasma Ferulic Acid Level Detection

**FIGURE 5:**
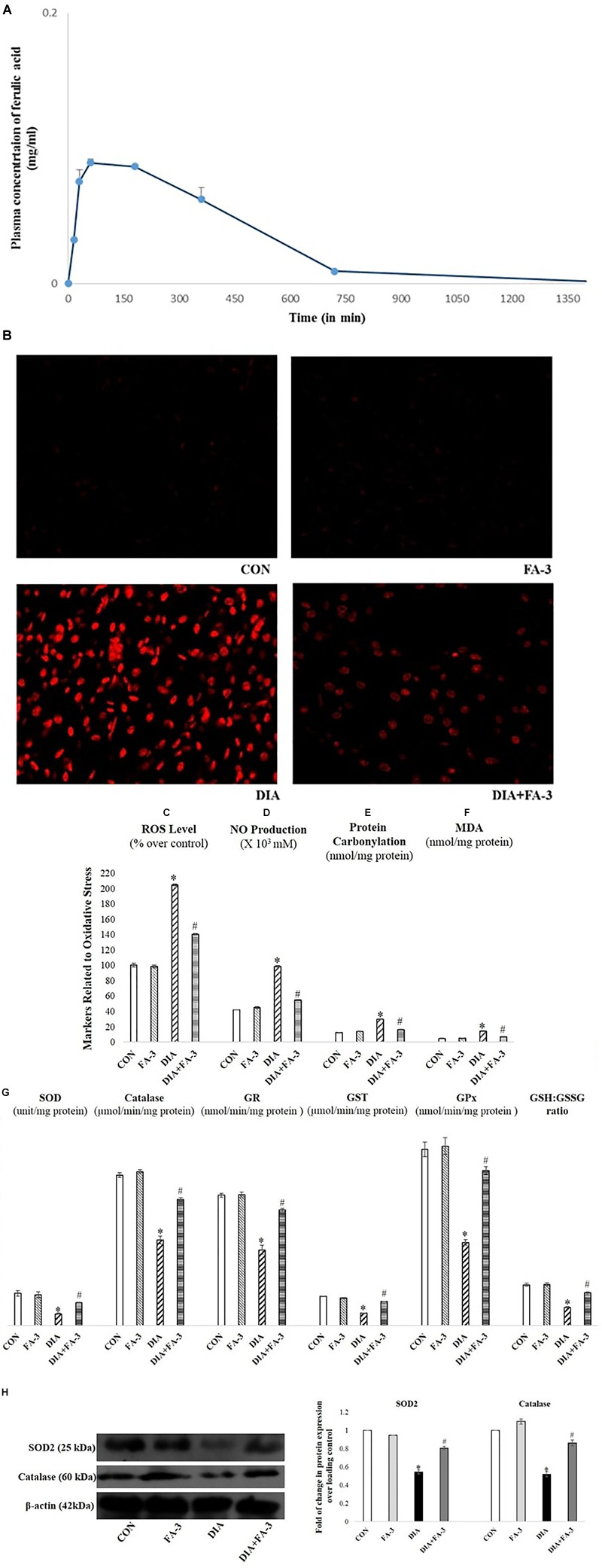
**(A)** Mean plasma concentration-time profiles succeeding oral administration of ferulic acid (50 mg kg^-1^ body wt.); each point represents the mean ± SD. Role of ferulic acid on parameters related to oxidative insult. **(B)** Detection of ROS by DHE staining (×400); **(C)** ROS level; **(D)** NO production; **(E)** protein carbonylation; **(F)** lipid peroxidation (MDA); **(G)** activities of antioxidant enzymes and assessment of redox ratio (GSH/GSSG); **(H)** assessment of SOD2 and catalase expression through western blot analysis and respective densitometry analysis of the same in the kidney tissue of the experimental rats. CON: untreated rats; FA-3: rats were subjected to ferulic acid treatment only; STZ: diabetic control; STZ+FA-3: rats administered with ferulic acid, post diabetes. Densitometry analysis are denoted as the mean ± SEM of three independent experiments, ^∗^*P* < 0.05 vs. CON; ^#^*P* < 0.05 vs. DIA; no significant difference existed between CON and FA-3 groups.

The recovery of ferulic acid over time from the plasma concentrations was analyzed by HPLC using a UV absorbance detector at 320 nm. HPLC analysis confirmed the absorption of the molecule in the blood of experimental rats (Figure 5A).

### Ferulic Acid Combats Excessive Production of ROS; Oxidative Insult-Associated Markers and Restores Cellular Antioxidant Capacity in STZ-Exposed Kidney Tissue

In the present study, the production of free radicals in the diabetic rats increased significantly compared to the untreated rats, thereby confirming the involvement of superoxide radical whereas; administration with ferulic acid post-diabetes could significantly (*p* < 0.05) decrease the free radical production (Figure 5B). Increased intracellular ROS level, NO production, elevated oxidative stress-related markers (protein carbonyl content and MDA, the final product of lipid peroxidation) and altered antioxidant activities express the state of intracellular oxidative stress (Sinha et al., 2013). The kidney tissue of hyperglycemic rats showed increased intracellular ROS (Figure 5C) and NO (Figure 5D) levels, enhanced protein carbonyl (Figure 5E) and MDA (Figure 5F) levels, decreased activities of antioxidant enzymes (Figure 5G), reduced expression of SOD2 and catalase (Figure 5H) and impaired cellular redox balance (by diminishing GSH/GSSG ratio) (Figure 5G). However, ferulic acid could significantly restore these altered parameters to the normal level (Figure 5B–H; *p* < 0.05), thereby reflecting the antioxidant capacity of the molecule against oxidative stress under diabetic complications.

### Ferulic Acid Reduces STZ-Induced Increased Advanced Glycation End Products (AGEs) -Expressions as Well as Xanthine Oxidase and Hydroxyproline Levels

**FIGURE 6:**
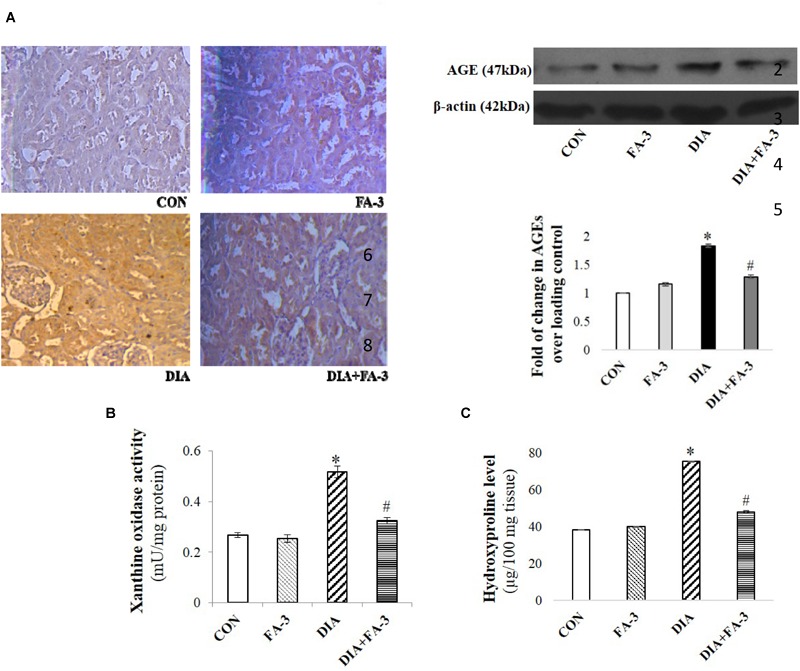
Effects of ferulic acid on STZ-mediated AGEs expression as well as hydroxyproline levels and xanthine oxidase. **(A)** IHC micrograph (×200) as well as western blot and densitometric analysis of the same showing changes in the expression of AGEs over different experimental groups; **(B)** xanthine oxidase level and **(C)** hydroxyproline content. Data represent mean ± SEM of three different experiments; “^∗^” indicates a significant difference between DIA and CON, whereas; “#” indicates a significant difference between DIA and DIA+FA-3; ^∗^*P* < 0.05 vs. CON; ^#^*P* < 0.05 vs. DIA; no significant difference existed between CON and FA-3 groups.

AGEs and xanthine oxidase are markers of ROS inducer under diabetic pathophysiology whereas hydroxyproline content correlates to fibrosis. From immunoblot data as well as immunohistochemistry, the diabetic group showed decreased AGEs expression concerning the control or only ferulic acid-treated group (*p* < 0.05) (Figure 6A). Also, the activities of renal xanthine oxidase (Figure 6B) and hydroxyproline level (Figure 6C) increased meaningfully in STZ-induced diabetic rats. Treatment with ferulic acid, however, suppressed AGEs formation, inhibited the xanthine oxidase activity and reduced the hydroxyproline content (Figure 6A–C; *p* < 0.05), thereby suggesting that the molecule could effectively block the activities of these ROS inducers as well as prevent renal fibrosis in diabetic rodents.

### Ferulic Acid Inhibits Hyperglycemia-Mediated MAPK Activation

**FIGURE 7:**
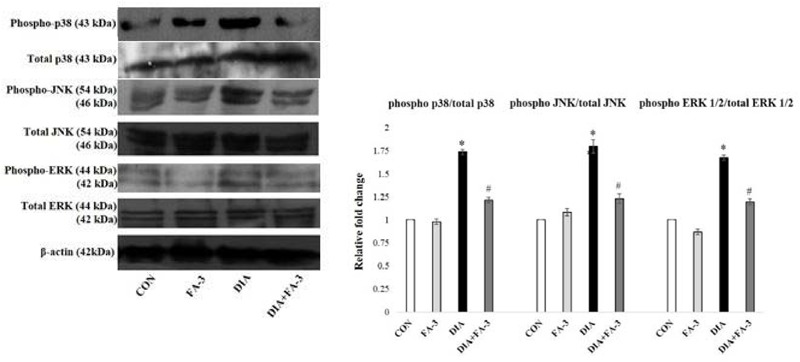
MAPKs activation in the STZ-mediated diabetic rats and effect of ferulic acid. Immunoblot analysis of phospho and total p38, phospho and total JNK, phospho and total ERK 1/2 and the densitometric analysis of the same. CON: only vehicle was given; FA-3: only ferulic acid was given; DIA: a single intraperitoneal dose of STZ was given; (DIA+FA-3): post-diabetes, treated with ferulic acid. Densitometry signifies mean ± SEM of three independent experiments, ^∗^*P* < 0.05 vs. CON; ^#^*P* < 0.05 vs. DIA; no significant difference existed between CON and FA-3.

Hyperglycemia triggered oxidative insult leads to the activation of MAPKs. Under varied pathophysiological conditions, the MAPK family proteins serve as the key inducers of apoptotic cell death (Das and Sil, 2012). To determine the effect of ferulic acid on the activation of MAPK families under hyperglycemic conditions, we performed immunoblotting for both phosphorylated and total forms of p38, JNK and ERK1/2 MAPKs. As observed from the data, ferulic acid treatment, post diabetes, significantly reversed (*p* < 0.05) the phosphorylation of p38, JNK and ERK1/2 MAPKs in the kidney tissue of STZ-mediated diabetic animals (Figure 7).

### Ferulic Acid Inhibits Neutrophil Infiltration in STZ-Mediated Damaged Renal Tissue

**FIGURE 8:**
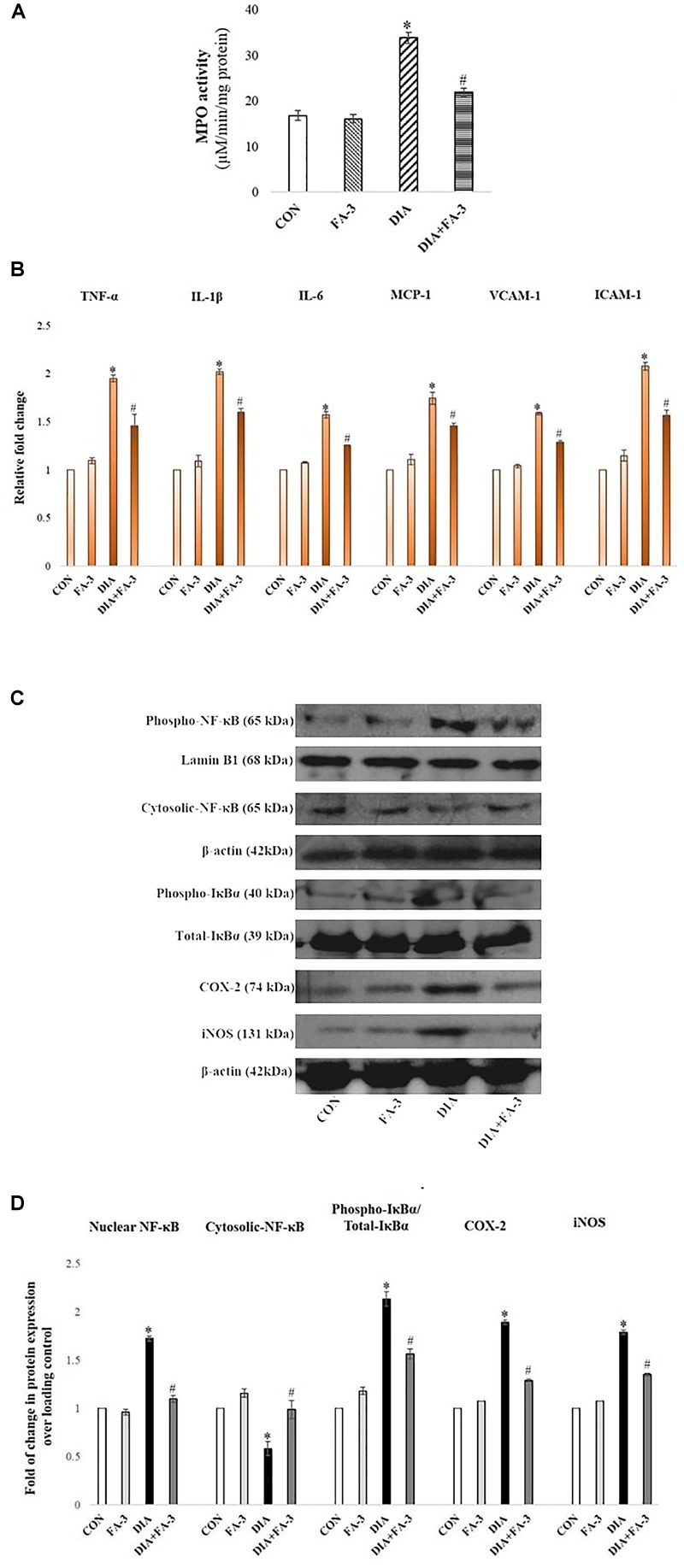
Role of STZ and ferulic acid on MPO activity and NF-κB mediated inflammatory pathways. CON: vehicle treatment alone; FA-3: treatment with only ferulic acid; DIA: a single intraperitoneal dose of STZ administration; (DIA+FA-3): post-diabetic induction, treatment with ferulic acid. **(A)** MPO activity analyses in the kidney tissues of different experimental groups; **(B)** Real-time PCR of cytokines (TNF-α, IL-1β, IL-6), chemokines (MCP-1) and adhesion molecules (VCAM-1, ICAM-1) were executed in triplicate and the results were represented as the fold differences of target gene expression relative to that of the loading control; **(C)** Immunoblot and densitometric analyses of nuclear and cytosolic NF-κB, phospho- and total IκBα, COX-2 and iNOS in the kidney tissue of experimental animals. Data are signified as the mean ± SEM of three different experiments; no significant difference existed between CON and FA-3 groups; ^∗^*P* < 0.05 vs. CON; ^#^*P* < 0.05 vs. DIA.

At the inflammation site, neutrophil infiltration is regulated by chemokines, cytokines, and adhesion molecules (Sinha et al., 2015). To quantitatively assess the infiltration of neutrophil at the tissue injury site, MPO activity was determined (Mullane et al., 1985). A significant elevation in MPO activity was observed in the diabetic rats (*p* < 0.05) as compared to the untreated set of animals, whereas; ferulic acid down-regulated (*p* < 0.05) the activity in the STZ-exposed diabetic rats compared to the only diabetic rats significantly (Figure 8A).

### Ferulic Acid Reduces Renal Inflammatory Cytokines, Chemokine and Adhesion Molecules

Exposure to STZ results in the upregulation of some proinflammatory cytokines, chemokine and various adhesion molecules in the renal tissue (Duran-Salgado and Rubio-Guerra, 2014). In our present findings, a marked elevation in the expression of proinflammatory cytokines (viz. TNF-α, IL-1β, and IL-6), chemokine (MCP-1)and adhesion molecules (ICAM-1 and VCAM-1) in the kidney tissue of STZ-exposed rats compared to the control rats (vehicle-treated) was confirmed by real-time PCR analysis (Figure 8B, *p* < 0.05). Ferulic acid, however, significantly reduced (*p* < 0.05) these expressions to a considerable extent showing its anti-inflammatory property in hyperglycemia-mediated renal tissue damage.

### Ferulic Acid Inhibits IκBα Degradation and Activation of the NF-κB-Mediated Pathway in STZ-Induced Renal Injury

As observed, cytosolic NF-κB expression was decreased, whereas the expression of IκBα, as well as expression nuclear NF-κB in the diabetic group, were increased. At the downstream of NF-κB, a significant increase in the level of iNOS and COX-2 was observed in the same group (Figure 8C; *p* < 0.05). Administration of ferulic acid, however, significantly altered the above mentioned adverse effects (Figure 8C; *p* < 0.05).

### Administration of Ferulic Acid Enhances Autophagy Induction in the Kidneys From STZ-Induced Diabetic Rats

**FIGURE 9:**
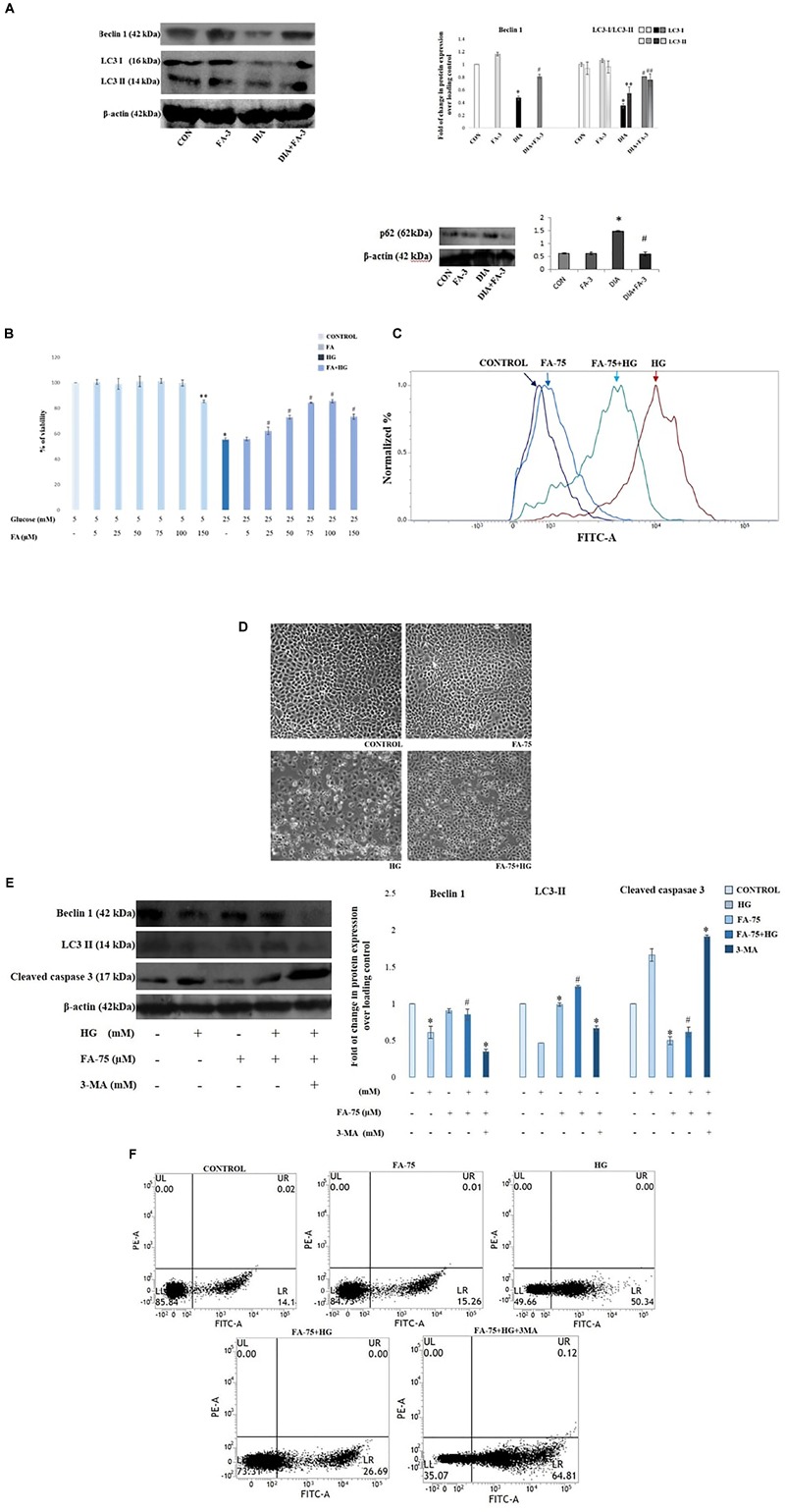
Effect of ferulic acid on autophagy flux and oxidative insult-mediated apoptosis in hyperglycemia-induced nephrotoxicity in diabetic rats as well as high glucose-induced NRK-52E cells. CON: treated with vehicle only; FA-3: treatment with ferulic acid only; DIA: administration with a single STZ exposure; DIA+FA-3: treatment with ferulic acid following diabetic induction. **(A)** Western blot and densitometric analyses of beclin-1, LC3 II and p62 expressions in the kidney tissue of experimental rats; **(B)** Dose-dependent change in viability (represented in % over control) in NRK-52E cells following pretreatment with ferulic acid (0-150 μM) for 2 h and successive exposure to 25 mM of glucose for 48 h. CONTROL: refers to the vehicle (water) treated control group; HG: cells exposed to high glucose, i.e., 25 mM of glucose for 48 h; FA-75+HG: pre-incubation with 75 μM ferulic acid for 2 h followed by high glucose (25 mM) introduction for next 48 h; FA-75: incubation with only 75 μM of ferulic acid; FA-75 + HG + 3-MA: inhibitor exposed NRK-52E cells. **(C)** Detection of endogenous ROS level in NRK-52E cells; **(D)** phase contrast micrographs (×100) for different experimental groups depicting external morphology of cells; **(E)** Immunoblot analysis as well as densitometric analysis reflecting the expression of beclin-1, LC3 II and cleaved caspase 3 following autophagy inhibition by 3-MA; **(F)** FACS analyses with Annexin V staining to determne the percent of apoptotic cells in the different experimental groups. Quadrants: lower left represents live cells; lower right represents apoptotic cells; upper left represents necrotic cells. Data have been expressed as the mean ± SEM for three different experiments; ^∗^*P* < 0.05 vs. control group; ^#^*P* < 0.05 vs. DIA/HG; ^∗∗^*P* < 0.05 vs. CONTROL; ^##^*P* < 0.05 vs. HG; HG, high glucose.

Immunoblot assay showed that autophagy-related proteins beclin-1 and LC3-II expressions were significantly decreased while expression of p62 increased in the kidney lysates of diabetic rats compared to the only vehicle-treated control animals; whereas ferulic acid significantly altered the STZ-induced impaired protein expressions (Figure 9A; *p* < 0.05).

### Ferulic Acid Induces Autophagy in High Glucose-Induced Cultured NRK-52E Cells

To further validate the role of ferulic acid associated with the induction of autophagy *in vivo* under the hyperglycemic condition, an *in vitro* inhibitor study was performed. Before the inhibitor study, the protective effect of ferulic acid in NRK-52E against high glucose-induced oxidative stress-mediated cell death was investigated.

#### Ferulic Acid Recovers Mitochondrial Dysfunction and Imparts Protection to NRK-52E Cells Against High Glucose-Induced Cell Death

Mitochondrial dehydrogenases (an important machinery of the electron transport chain) are prone to ROS-triggered damage (Orrenius et al., 2007). Exposure to 25 mM glucose reduced the activity of mitochondrial dehydrogenases in NRK-52E cells, whereas; ferulic acid-treatment significantly recovered the activity in a dose-dependent manner, the optimum dose of protection being 75 μM (Figure 9B; *p* < 0.05). High glucose-induced oxidative insult is a pathophysiological state of the cells. The effect of ferulic acid on endogenous ROS production was investigated. The intracellular ROS accumulation in high glucose-exposed cells increased to a great extent compared to the untreated group and significantly decreased on treatment with ferulic acid as evident from DCF fluorescence intensity (Figure 9C). Furthermore, high glucose-mediated cells showed characteristics of stress and apoptosis viz. losing normal morphology of cells, membrane blebbing, membrane detachment and formation of apoptotic bodies. However, ferulic acid could significantly restore such cellular morphological changes (Figure 9D).

After that, the inhibitor study was conducted to confirm the role ferulic acid in autophagy induction. When subjected to autophagy inhibitor (3-MA) pre-treatment (at a concentration of 5 mM, 1 h), the expression levels of beclin-1 as well as LC3-were inhibited. Thus, from the observations above, it can be inferred that inhibition of autophagy abolishes the ameliorative role of ferulic acid against high glucose-induced renal damage (Figure 9E). Furthermore, flow cytometric analysis revealed that the percentage of dead cells markedly increased in high glucose-exposed cells, while pre-incubation of ferulic acid could significantly diminish high glucose-triggered cell apoptosis. However, following 3-MA pre-treatment, the expression level of the effector caspase (caspase 3) was elevated (Figure 9E) as well as stimulate tubular cell apoptosis (Figure 9F). Thus, it can be concluded that inhibition of autophagy upregulated apoptosis.

### Ferulic Acid Prevents Apoptotic Cell Death With the Down-Regulation of Mitochondria-Dependent Signaling Cascade as Well as Extracellular Death Signal Cascade in STZ-Exposed Renal Tissue

**FIGURE 10:**
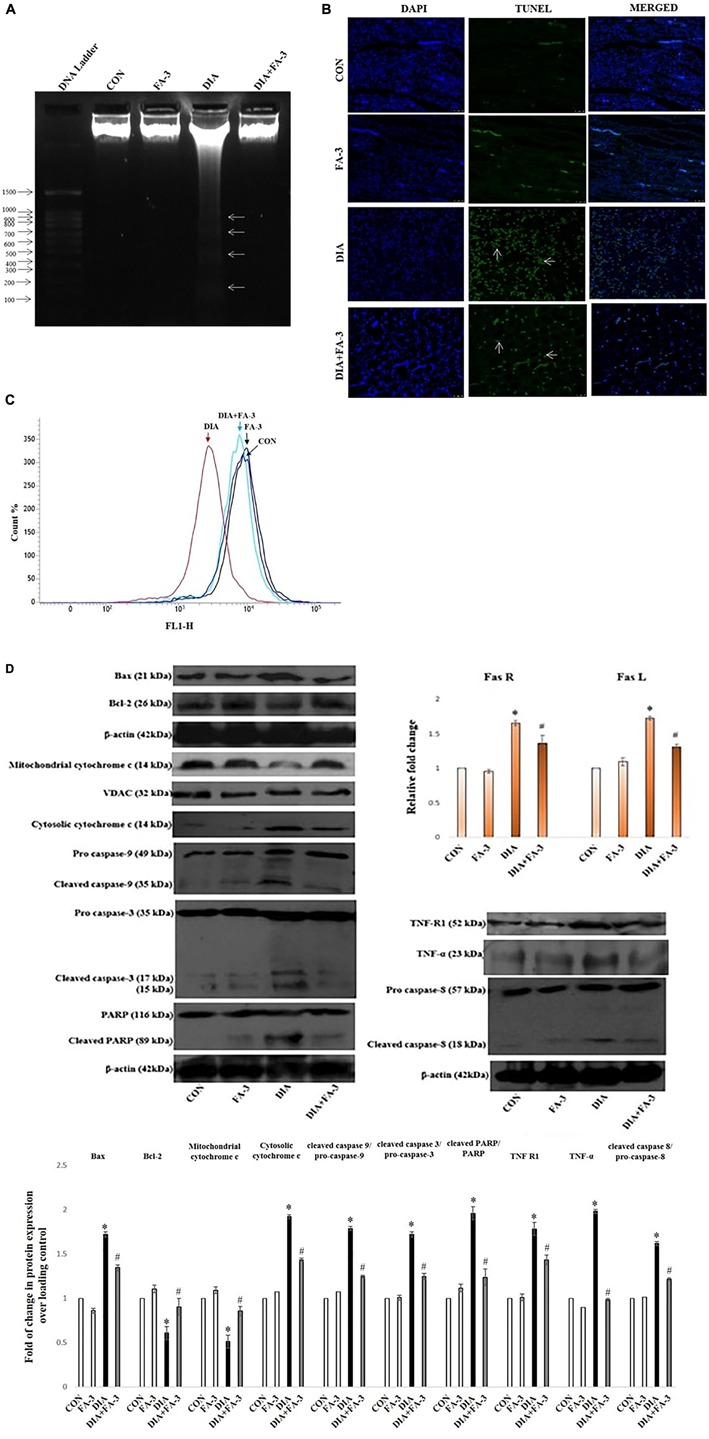
Effect of ferulic acid on both mitochondria-dependent and -independent manner of cell death in hyperglycemia-induced renal injury. CON: untreated control rats, FA-3: rats were subjected to ferulic acid (50 mg kg^-1^ body wt.) treatment only, DIA: diabetic control group, DIA + FA-3: diabetic rats were subjected to ferulic acid (50 mg kg^-1^ body wt.) treatment. Mode of cell death: **(A)** DNA fragmentation assay on agarose/EtBr gel 1.8% (w/v) (arrows symbolises DNA fragments); **(B)** TUNEL assay of the kidney tissue of experimental rats. TUNEL-positive splenic cells are stained in green and the nuclei are stained in blue (with DAPI; ×400); **(C)** MMP analysis of different experimental groups by FACS; **(D)** Western blot and densitometric analyses of Bax, Bcl-2, mitochondrial cytochrome c, cytosolic cytochrome c, caspase 9, caspase 3, PARP, TNF R1, TNF-α, caspase 8 as well as real time PCR of Fas R and Fas L. Data are represented as the mean ± SEM for three independent experiments; ^∗^*P* < 0.05 vs. control group; ^#^*P* < 0.05 vs. diabetic group.

To observe the mode of cell death, agarose gel electrophoresis was performed. Characteristic DNA ladder, a symbol of apoptosis was observed in the diabetic animals, whereas; ferulic acid could significantly recover DNA fragmentation (Figure 10A). Furthermore, TUNEL assay was performed to detect the apoptotic index. In STZ-induced diabetic kidney tissue of rats, TUNEL positive nuclei staining was observed, thus confirming apoptosis. However, ferulic acid helped in the diminution of TUNEL positive staining of the nuclei in the kidney tissue of diabetic animals (Figure 10B).

Next, we determined the role of mitochondria to induce apoptosis because mitochondrial oxidative stress and induction of apoptosis are interrelated. Flow cytometry data confirmed that MMP reduced significantly in the diabetic rats while post-treatment with ferulic acid significantly elevated the reduction (Figure 10C).

Immunoblot analysis showed downregulation in the level of mitochondrial cytochrome c and up-regulation in the levels of cytosolic cytochrome c, Bax/Bcl-2 ratio and cleaved caspase 9 in the diabetic rats (Figure 10D). Also, the involvement of the extrinsic pathway in STZ-triggered apoptosis was also confirmed by measuring the expression of Fas-L, Fas-R, TNF-α and caspase-8 (crucial factors involved in the extrinsic pathway of apoptosis) (Figure 10D). Real-time PCR analysis showed an increase in the levels of Fas-L and Fas-R, whereas; immunoblot analysis showed caspase-3 activation and PARP cleavage in diabetic rats (Figure 10D). However, ferulic acid decreased the expression of apoptotic markers under the hyperglycemic condition, thus proposing its potential anti-apoptotic property in suppressing both the mitochondria-dependent and independent apoptotic pathways (Figure 10D).

## Discussion

Existing literature suggests the contribution of oxidative stress in the pathogenesis of diabetic nephropathy (Vasavada and Agarwal, 2005). Under the hyperglycemic condition, the redox balance shifts toward a pro-oxidant state which in turn triggers tissue and vascular injury. Hence, strategies to combat oxidative stress regarding intervention for hyperglycemia-induced renal damage were explored.

In this study, the beneficial role of ferulic acid, to combat diabetes-induced adverse effects on kidney tissue, was investigated and we have discussed that (i) ferulic acid treatment significantly mitigated hyperglycemia-mediated renal injury, as assessed by morphological and histopathological measures as well asby the biochemical analysis of serum renal markers viz. creatinine, BUN etc. and key markers of diabetic pathophysiology viz. blood glucose level, insulin, etc. (ii) administration of ferulic acid reduced oxidative stress under diabetic condition (*in vivo*) and high glucose environment (*in vitro*) by enhancing the activity of antioxidants and combating ROS generation; (iii) circumvented the hyperglycemic pathophysiology by regulating MAPK activation and AGEs expressions; (iv) induced otherwise inhibited autophagy *in vivo* under diabetic status, reduced p62 expression indicating amelioration of defects in autophagic pathway as well as improved defective basal autophagy inhibition by 3-MA under high glucose environment in cultured NRK-52E cells; (v) decreased urinary albumin excretion and mitigated glomerulosclerosisby regulating NF-κB-triggered inflammatory molecules; and (vi) inhibited cell apoptosis under diabetic hyperglycemia in rats as well as high glucose environment in NRK-52E cells.

The free radical scavenging ability of ferulic acid is dependent on the presence of hydroxyl groups in the benzene ring as well as ortho substitution with the electron donor methoxy group which increases the resonance stability of the phenoxy radical (Kikuzaki et al., 2002; Sinha et al., 2007; Das et al., 2009; Bhattacharya et al., 2013; Ghosh and Sil, 2013). In the present study, a significant increase in the levels of the creatinine, BUN, urinary albumin and uric acid content is in accordance with the existing literature suggesting progressive nephrotoxicity in hyperglycemic rats. Besides, histological studies showed glomerulosclerosis, glomerular hypertrophy and tubulointerstitial damage in STZ-administered diabetic animals (Das and Sil, 2012). Scarring of glomeruli interferes with the kidneys’ filtering process which results in protein leakage from the blood into the urine (a condition termed as proteinuria). A complex interplay between the extracellular matrix and factors in the tubular lumen, tubular epithelial cells, infiltrating interstitial cells and peritubular capillaries results in tubulointerstitial damage (Das and Sil, 2012). In the present study, ferulic acid significantly reduced the increased levels of renal damage markers as well as glomerular hypertrophy in addition to the restoration of blood glucose and insulin levels.

Literature suggests that under the hyperglycemic condition, production of excessive ROS and NO results in oxidative insult to intracellular protein as well as ruptures the membrane-bound phospholipids through membrane lipid peroxidation (Sarkar and Sil, 2006; Manna et al., 2009, 2010; Kashihara et al., 2010; Das and Sil, 2012; Das et al., 2012; Pal et al., 2014; Rashid and Sil, 2015a,b; Rashid et al., 2017). As observed, treatment with ferulic acid, could effectively combat ROS production as well as restore protein carbonylation, lipid peroxidation, and antioxidant activities.

A significant elevation in hydroxyproline content was observed in STZ-induced diabetic animals which contribute to the severity of kidney fibrosis and lesions in subjects suffering from diabetes. Ferulic acid, however, effectively diminished the anomaly. Hyperglycemia contributes to AGEs formation (reactive intracellular dicarbonyls react with the amino groups of intracellular and extracellular proteins to form AGEs (Brownlee, 2001) which is present in diabetic renal glomeruli according to the literature (Horie et al., 1997) as well as increase xanthine oxidase activity. As observed, ferulic acid significantly suppressed the AGEs formation and inhibited xanthine oxidase activity in STZ-mediated diabetic renal tissue.

MAPK cascades (p38 MAPK, c-Jun N-terminal kinase/stress-activated protein kinase or JNK/SAPK and ERK 1 and 2 or p44/p42 MAPKs) have been extensively reported to be involved in hyperglycemia mediated extracellular matrix accumulation in diabetic nephropathy (Brosius et al., 2010). From our findings, we have also demonstrated that the protein expression levels of phospho-p38 MAPK, phospho-JNK and phospho-ERK1/2 were upregulated in the renal tissues of STZ-mediated diabetic rats, whereas; ferulic acid ameliorated ROS-induced upregulation of MAPKs.

In our study, we observed impaired autophagy due to decreased expression of beclin-1 and LC3-II in the diabetic group. However, such alterations were mitigated through treatment of ferulic acid. p62 serves as a connection between LC3 and ubiquitinated substrates in course of their journey toward the autophagosomes and thus can be used to monitor of autophagic flux. Therefore, accumulation of p62 indicates defect in the autophagic pathway (Deshpande et al., 2018). In our study, increased expression of p62 in the diabetic group was indicative of impaired autophagy while decrease in its expression following treatment with ferulic acid pointed toward restoration of normal autophagic pathway.

Increased free radical generation and impaired autophagy participate in the apoptotic death of renal cell under hyperglycemic condition (Pal et al., 2014). In apoptosis, caspase–activated DNases degrades DNA which is in accordance with our findings. Initiation of the extrinsic cascade of apoptosis involving the TNF-α binding with its receptor TNF-R1 as well as dysregulation of the Fas/Fas ligand system caused activation of caspase-8 which favors apoptotic cell loss in diabetes (Maedler et al., 2001). STZ-induced apoptosis has also been reported to trigger the intrinsic mitochondrial pathway with the oligomerization of Bax/Bak in mitochondria and subsequent release of cytochrome c in the cytosol (Wu et al., 2011; Pal et al., 2014). In the present study, ferulic acid inhibited both mitochondria-dependent and -independent apoptosis by downregulating STZ induced elevated FasL/FasR, TNF-α/TNF-R1, caspases (8, 9, 3) and PARP expressions under the diabetic condition, thus combating apoptosis in rat kidney tissue.

Our extensive study of the ameliorative role of ferulic acid against hyperglycemia induced renal dysfunctions is indicative of its candidacy to act as a potential therapeutic. However, a more detailed insight into the molecular mechanisms indicated in this study is essential to conform to further proceedings in course of its journey toward a component of prescribed medications.

## Conclusion

**FIGURE 11:**
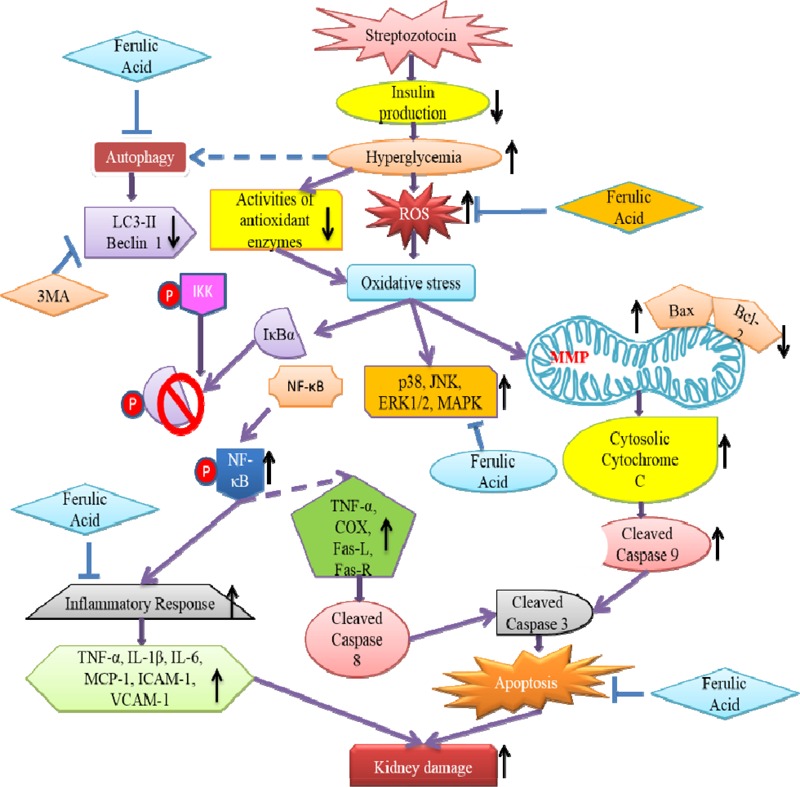
Schematic representation indicating the probable mechanisms through which ferulic acid provides protection against streptozotocin-triggered hyperglycemia-induced renal damage in rats; (“blunt arrows”: inhibitory interaction; “pointed arrows”: stimulatory interaction; “dotted arrows”: probable mechanisms).

The study sheds light into the probable molecular mechanism by which ferulic acid, exerting its antioxidant, hypoglycemic, anti-inflammatory, anti-apoptotic as well as autophagic activities, can significantly combat diabetes-associated renal complications (Figure 11).

## Author Contributions

All authors listed have made a substantial, direct and intellectual contribution to the work, and approved it for publication.

## Conflict of Interest Statement

The authors declare that the research was conducted in the absence of any commercial or financial relationships that could be construed as a potential conflict of interest.

## Abbreviations

AGE: advanced glycation end product
BUN: blood urea nitrogen
DCFDA: dichloro fluorescein diacetate
DHE: dihydroethidium
DMEM: Dulbecco’s modified Eagle medium
DNPH: dinitro phenyl hydrazine
DPPH: 2,2-diphenyl 1-picryl hydrazyl
EDTA: ethylene diamine tetracetate
EGTA: ethylene glycol tetracetate
ELISA: enzyme linked immunosorbent assay
ERK: extracellular signal regulated kinase
FITC: fluorescein isothiocyanate
FRAP: ferric reducing antioxidant power
GPx: glutathione peroxidase
GSH/GSSG: glutathione reduced/oxidized
GR: glutathione reductase
GST: glutathione S-transferase
H&E: hematoxylin and eosin
HPLC: high performance liquid chromatography
IL-6: interleukin-6
JNK: Janus kinase
MAPK: mitogen activated protein kinase
MCP-1: monocyte chemotactic protein-1
MDA: malondialdehyde
MMP: mitochondrial membrane potential
MPO: myeloperoxidase
NF-κB: nuclear factor kappa B
NO: nitric oxide
NOS: nitric oxide synthase
PARP: Poly-ADP ribose polymerase
ROS: reactive oxygen species
SAPK: stress activated protein kinase
SGOT: serum glutamic oxaloacetic transaminase
SGPT: serum glutamic pyruvic transaminase
SOD: superoxide dismutase
TBARS: thiobarbituric acid reactive substance
TNF-α: tumor necrosis factor-α
TUNEL: terminal deoxynucleotidyl transferase dUTP nick end labeling
